# Regulation of antibody responses against self and foreign antigens by Tfr cells: implications for vaccine development

**DOI:** 10.1093/oxfimm/iqab012

**Published:** 2021-06-16

**Authors:** Afonso P Basto, Luis Graca

**Affiliations:** CIISA—Centro de Investigação Interdisciplinar em Sanidade Animal, Faculdade de Medicina Veterinária, Universidade de Lisboa, Lisboa, Portugal; Instituto de Medicina Molecular, Faculdade de Medicina, Universidade de Lisboa, Lisboa, Portugal; Instituto de Medicina Molecular, Faculdade de Medicina, Universidade de Lisboa, Lisboa, Portugal; Instituto Gulbenkian de Ciência, Oeiras, Portugal

**Keywords:** T follicular regulatory cells, T follicular helper cells, vaccines, antibodies, germinal centres, autoimmunity

## Abstract

The production of antibodies can constitute a powerful protective mechanism against infection, but antibodies can also participate in autoimmunity and allergic responses. Recent advances in the understanding of the regulation of germinal centres (GC), the sites where B cells acquire the ability to produce high-affinity antibodies, offered new prospects for the modulation of antibody production in autoimmunity and vaccination. The process of B cell affinity maturation and isotype switching requires signals from T follicular helper (Tfh) cells. In addition, Foxp3^+^ T follicular regulatory (Tfr) cells represent the regulatory counterpart of Tfh in the GC reaction. Tfr cells were identified one decade ago and since then it has become clear their role in controlling the emergence of autoreactive B cell clones following infection and immunization. At the same time, Tfr cells are essential for fine-tuning important features of the humoral response directed to foreign antigens that are critical in vaccination. However, this regulation is complex and several aspects of Tfr cell biology are yet to be disclosed. Here, we review the current knowledge about the regulation of antibody responses against self and foreign antigens by Tfr cells and its implications for the future rational design of safer and more effective vaccines.

## INTRODUCTION

The induction of long-lasting high-affinity antibodies is a major goal in vaccination and underlies the effectiveness of most protective vaccines developed so far [1]. On the contrary, vaccine-induced antibodies with low affinity, or inadequate antibody isotypes, may not only render vaccines ineffective but can also contribute to vaccine-associated immunopathology [2]. Dysregulated antibody responses are also implicated in the pathophysiology of many autoimmune and allergic diseases. Therefore, a greater understanding of the mechanisms driving and regulating the production of high-affinity antibodies will certainly contribute for a better control of immune-mediated disorders and for the development of safer and more effective vaccines.

The plasma and memory B cells responsible for the production of high-affinity antibodies are generated in microanatomical structures—named germinal centres (GCs)—that are formed within the B cell follicles of secondary lymphoid tissues upon antigenic stimulation [3]. The role of CD4^+^ T cells in providing B cell help for antibody production is a long-established knowledge in immunology [4]. However, the way we perceive the T–B cell interactions underlying this process was revolutionized in the last two decades with the identification of specialized effector and regulatory CD4^+^ T cell subsets that, contrary to classical T helper cells, enter the B cell follicles and the GCs—the T follicular helper (Tfh) [5] and T follicular regulatory (Tfr) cells [6, 7].

Tfh cells are primed by dendritic cells (DCs) in the T-cell areas of secondary lymphoid organs upregulating the chemokine receptor CXCR5 and Bcl6—the lineage-defining transcription factor of Tfh cells [8–10]. CXCR5 expression facilitates the migration of pre-Tfh cells towards the T–B border where the interaction with cognate B cells reinforces their follicular genetic programme [5]. This reinforcement leads to Tfh migration into the GC where Tfh cells play an essential role for the selection of B cell clones producing high-affinity antibodies and for the differentiation of long-lived plasma and memory B cells [5].

**Figure 1:**
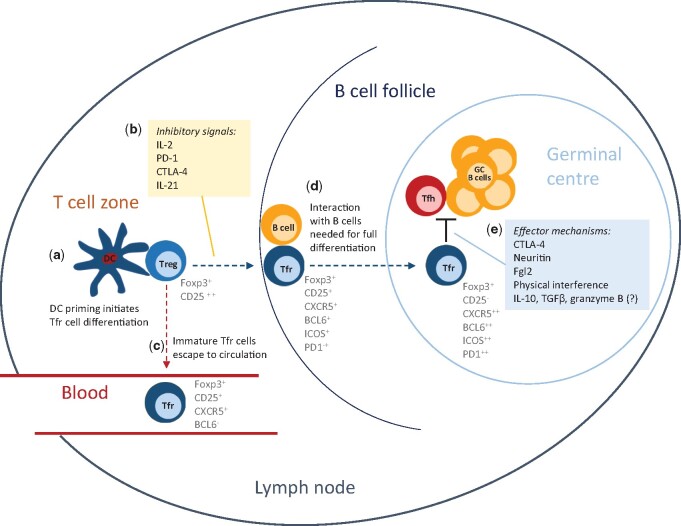
differentiation, effector mechanisms and phenotypic markers of Tfr cells. Tfr cells differentiate from naive Foxp3^+^ Treg cells upon DC priming at the T cell zone of secondary lymphoid tissues (**a**). The soluble and contact-dependent factors favouring Tfr differentiation are yet to be discovered but signalling via the surface molecules PD-1 and CTLA-4 and the cytokines IL-2 and IL-21 have been shown to play an inhibitory role in Tfr differentiation (**b**). Once primed, Tfr cells can either escape to blood circulation maintaining an immature state (**c**) or, upon interaction with B cells, proceed towards complete maturation as they move towards the GC (**d**). In the GC, Tfh cells provide help and select GC B cell clones with mutated BCR genes leading to the production of high-affinity antibodies. This reaction is tightly regulated by Tfr cells through effector mechanisms that include CTLA-4 blocking of CD80/CD86 co-stimulation; secretion of effector molecules that directly act on B cells and/or Tfh (e.g. neuritin and Fgl2); physical interference on Tfh–GC B cell interactions; other not yet identified or poorly characterized mechanisms (**e**). The phenotypic markers defining Tfr cells at the different stages are shown.

Tfh-driven GC reactions are controlled by Tfr cells, a specialized regulatory T cell population identified 10 years ago [11–13]. Tfr cells express simultaneously the CXCR5 chemokine receptor, that confers access to the B cell follicle, and the canonical regulatory transcription factor Foxp3, thus representing the regulatory counterpart for Tfh cells in the GC reaction [6, 7, 14] (Fig. 1). Tfr cells control the emergence of self-reactive antibodies, preventing antibody‐mediated autoimmunity and regulate the response against foreign antigens, thereby contributing for a fine-tunned affinity maturation of antibody responses [6, 7].

Since their discovery, Tfh and Tfr cells became appealing targets both for therapeutic interventions to tackle antibody-mediated disorders and for improving vaccine-induced responses. This review discusses the present knowledge on the Tfr regulation of antibody responses directed towards self and foreign antigens, focusing on the implications for the development of safer and more effective vaccines.

## REGULATION OF SELF-REACTIVE ANTIBODY RESPONSES BY TFR CELLS

Mice and humans devoid of functional Foxp3^+^ T regulatory cells develop a severe autoimmune syndrome that includes uncontrolled humoral immunity [15–19]. It is thus not surprising that when a specialized Treg subset with unique access to B cell follicles was discovered, its specific role in controlling autoantibody responses was hypothesized [11–13]. In fact, a major feature of GC reactions is providing the conditions for the emergence and selection of B cell clones with increasing affinity towards the foreign antigens that elicit the B cell response [3]. However, this process relies on the random somatic hypermutation of the B-cell receptor (BCR) genes, which may result in the emergence of self-reactive antibodies and ultimately lead to autoimmunity, thus requiring tight regulation [20].

Although the division of labour between non-follicular and follicular T regulatory cells in controlling this process is not yet fully elucidated [21], it is now clear that Tfr cells play a critical role in preventing the emergence of autoantibodies in GCs. This is supported by three main lines of evidence: first, the demonstration that Tfr cells preferentially derive from thymic Foxp3^+^ Tregs, with a self-biased T cell receptor (TCR) repertoire [11–13, 22–26]; secondly, the observation that a specific Tfr deletion in mouse models leads to the emergence of self-reactive antibodies and autoimmunity [23, 27–30]; finally, the growing number of human studies finding correlations between the manifestation of autoimmune diseases and quantitative or qualitative alterations in blood Tfr cells of autoimmune patients [31, 32].

### Tfr precursors and antigen-specificity

Mouse studies have shown that Tfr cells, as Tfh and GC B cells, arise in secondary lymphoid tissues upon infection or vaccination. We have also observed that Tfr cell numbers increase in the blood of healthy adults after influenza vaccination [22]. A central question to elucidate Tfr cell function, and understand their mode of action, was thus to clarify whether Tfr cells are specific to the foreign antigen that triggers their differentiation or, alternatively, preferentially recognize self-antigens. This issue directly relates with the question of whether Tfr cells derive from natural Tregs (which have a self-biased TCR repertoire) or from peripheral naive Tconv cells (which are specific for non-self antigens). The first three studies describing Tfr cells in 2011 investigated the Tfr ontogeny, and all pointed the thymic Foxp3^+^ cells as the main precursors of Tfr cells [11–13].

Linterman *et al.* [12] observed that, upon immunization, antigen‐specific TCR‐transgenic CD4^+^ cells, which do not contain thymic Treg cells, did not differentiate into Tfr cells when transferred into congenic wild-type (WT) mice. Moreover, when either Foxp3^+^ or Foxp3^−^ thymic CD4^SP^ T cells were transferred into congenic mice, only the Foxp3^+^ cells differentiated in Tfr cells upon sheep red blood cells (SRBC) immunization. Our group used T‐cell‐deficient mice (TCRα^−/−^) as recipients of antigen‐specific TCR‐transgenic CD4^+^ T cells and thymic Treg cells demonstrating that only the thymic Treg cells could differentiate into Tfr cells following immunization with ovalbumin (OVA)/alum [11]. Chung *et al.* also used T‐cell‐deficient (TCRβ^−/−^) mice as recipient, and co‐transferred WT naive CD4^+^ T cells and CXCR5^-^Foxp3^+^ Treg cells with different congenic markers, and showed that upon immunization with keyhole limpet haemocyanin (KLH) and complete Freund’s adjuvant (CFA) >98% of Tfr cells formed in the host mice originated from the CXCR5^−^Foxp3^+^ Treg cell population [13]. More recently, Botta *et al.* [23] have shown that unlike conventional Treg cells, which are CD25^hi^, Tfr cells are CD25^lo^, and thus tested whether Tfr cells derive from pre-existing CD25^hi^Foxp3^+^ or CD25^lo^Foxp3^+^ T cells. The authors quantified Tfr cells 30 days after influenza infection in mice that received congenically marked CD25^hi^Foxp3^+^ or CD25^lo^Foxp3^+^ cells and found significantly higher numbers in recipients of CD25^hi^Foxp3^+^ cells, thus corroborating the conclusions of previous works using an infection model. Our studies with human T cells also show that Tfr cells differentiate from Foxp3^+^ Treg cells within lymphoid tissue, originating Tfr cells that fully mature within GCs and more immature Tfr cells that enter the blood circulation [22, 26] (Fig. 1).

Although all these studies point the conventional Treg cells as the main precursors of Tfr cells, it has been shown that Tfr cells can also arise from peripherally induced Treg cells and be specific for the immunizing antigen, particularly in the absence of competition with thymic Treg cells and when specific adjuvants are used [33]. However, under physiological competition with thymic Treg cells the differentiation from induced Treg cells appears to be infrequent [24]. In fact, while following immunization it is possible to visualize Tfh cells specific for the immunizing antigen with an appropriate MHC class II tetramer, antigen‐specific tetramer‐positive Tfr cells are hardly detected [24]. Furthermore, the restimulation of sorted Tfr cells *ex vivo* with DCs loaded with immunizing antigen does not lead to preferential proliferation or survival when compared with a control antigen [24]. Finally, analysis of the TCR repertoire of Tfr, Tfh and Treg cell populations by sequencing the TCR*α*‐chain gene from immunized mice that have a fixed TCRβ chain shows that the Tfr pool has a TCR repertoire that more closely resembles the predominantly self‐reactive repertoire of thymic Treg cells [24]. This difference in the Tfh and Tfr repertoire was independently confirmed by Ritvo *et al.* [25], who have also shown, by TCR sequencing, that Tfh cell responses are predominantly focused on foreign antigens, while Tfr cell responses are mostly directed towards self-antigens.

Some studies reported that in mucosal-associated lymphoid tissues, or under inflammatory conditions, Foxp3^+^ Treg cells can be converted into Tfh cells [34–36]. Outside mucosal sites, it was also shown, using lineage tracking, that Tfr cells can lose Foxp3 expression, becoming ex-Tfr cells with diminished suppressive function [14]. When generated *in vitro*, these ex-Tfr cells lose the Tfr transcriptional programme and show a transcriptional signature more similar to Tfh cells [14]. Under these conditions, it is anticipated some degree of overlap between the repertoire of Tfh and Tfr cells, although the polarization of Tfh cells from naive precursors is likely to remain the predominant contribution to the overall Tfh pool given the smaller number of Tfr cells within the follicle.

### Autoreactive responses in mice devoid of functional Tfr cells

The demonstration that Tfr cells preferentially differentiate from thymic Foxp3^+^ cells, with a TCR repertoire potentially biased towards autoantigens, supports a model where Tfr cells play a role in preventing the generation of autoantibodies. A major advance for the validation of this concept was the recent development of murine models allowing the conditional depletion of Tfr cells by using Foxp3^Cre^ mice with floxed Bcl6 or Cxcr5 genes [37]. Indeed, these systems provided the necessary tools for the demonstration that the absence of Tfr cells is sufficient for the development of autoimmunity in mice [23, 27–30].

Taking advantage of the specific Tfr sensitivity to interleukin-2 (IL-2), Botta *et al.* first used rIL-2-treatment to deplete Tfr cells in influenza-infected mice and observed that the frequency of anti-histone IgG antibody-secreting cells (ASCs) increased in treated mice, suggesting that a lack of Tfr cells results in the development of anti-nuclear antibody responses. This was then confirmed in the same study using a Bcl6^fl/fl^Foxp3^YFP/Cre^ mice model in which influenza-infected mice have shown anti-nuclear antibodies 30 days post-infection [23].

In Bcl6-floxed systems, the emergence of autoantibodies was observed not only upon a deliberate infection but, in the long-term, also in the steady-state [27, 28]. Tfr-deficient mice have been shown to spontaneously produce anti-dsDNA, anti-nuclear antibodies, anti-salivary gland and anti-nuclear ribonucleoprotein/Smith antibodies at 30 weeks of age, and to develop inflammatory cell infiltrations in the lung, pancreas and salivary gland [27]. Gonzalez-Figueroa *et al.* [28] also detected increased levels of lupus-associated antibodies against extractable nuclear antigens in the plasma of 15-week-old mice and against histone H1 in 25-week-old, as well as autoantibodies directed to exocrine pancreas, stomach, salivary gland and the gastric parietal cell antigens.

The role of Tfr cells in the development of autoimmunity was further evaluated using the same Bcl6^fl/fl^Foxp3^Cre^ knockout (KO) strategy in autoimmune mouse models [27, 29]. In Sjögren’s syndrome model, Tfr-depleted mice have shown reduced saliva secretion, elevated serum autoantibodies and tissue destruction associated with lymphocytic infiltration in the submandibular gland [27]. In the same line, Tfr-deficient mice revealed elevated anti-dsDNA IgA serum antibodies in a pristane-induced lupus murine model, although arthritis signs were not identified in this study [29].

Vanderleyden *et al.* [38] developed a different KO strategy in which the Cxcr5 gene is deleted from Foxp3-expressing cells. Interestingly, although Treg cells expressing CXCR5 were successfully depleted with this system, microscopy studies have shown that Tfr cells, although at lower levels, were still found inside the GCs. In these settings, with about half the numbers of follicular and GC Tfr cells than the control mice, the levels of anti-dsDNA IgGs in the serum were unchanged after immunization with 4‐hydroxy‐3‐nitrophenylacetyl (NP)-KLH/alum.

Finally, using a murine model where cells that simultaneously express CXCR5 and Foxp3^+^ can be depleted by administration of diphtheria toxin (Tfr-DTR model), Clement *et al.* [30] observed that mice immunized with NP-OVA given DT before GC initiation has increased autoreactive IgG and IgE as detected by an autoantigen protein array. Interestingly, some of the autoantigens recognized by these antibodies are antigens targeted by self-reactive antibodies found in patients with systemic lupus erythematosus.

Together, by using different approaches to specifically deplete Tfr cells and address steady-state, immunizing and infection settings, the studies unequivocally ascribe a role for Tfr cells in preventing autoantibody responses and their pathologic consequences. This is corroborated by studies in murine models in which Tfr cells were not specifically depleted but where dysfunctional Tfr cells were implicated in the break of self-tolerance and consequent autoimmunity [39–42].

### Tfr cells in human autoimmune diseases

While in mice the specific manipulation of Tfr cells made evident their role in controlling antibody-mediated autoimmunity, the clarification of how Tfh and Tfr cells contribute to the course of autoimmune diseases in humans has been more difficult to establish. Due to the limited access to human lymphoid tissues, circulating Tfh and Tfr cells are often used as surrogates to assess GC activity. However, circulating Tfr and Tfh cells, although representing markers of ongoing humoral activity [22], do not necessarily reflect the alterations occurring in lymphoid and non-lymphoid tissues. Moreover, these studies have been using different strategies for the identification of circulating T follicular subsets, and the subclassification of relevant populations was not always performed (e.g. regulatory versus non-regulatory cells among CXCR5^+^ cells, or CXCR5^+^ versus CXCR5^−^ among Foxp3^+^ populations) [31, 32].

Another source of variability in human studies, and possible reason for discrepancies in the reported results, is the heterogeneity of the immunopathologic mechanisms or the distinct stages of disease among different patients diagnosed with the same disease. In fact, our group has shown that the activation status of Tfh cells and Tfr/Tfh ratio in the blood of Sjögren syndrome patients is associated with different features of the disease [43]. While an increased Tfr/Tfh ratio indicates the formation of ectopic lymphoid structures in target organs (namely, the minor salivary glands), the frequency of blood PD-1^+^ICOS^+^ Tfh cells correlates with disease activity [43]. These findings emphasize the need to take in account the disease heterogeneity when evaluating blood Tfh and Tfr cells subsets, but also open the possibility for stratification of patients with autoantibody-mediated diseases for a better therapeutical management.

Although these limitations make difficult the interpretation and comparison of results, a growing number of studies are reporting correlations between autoimmune disease and alterations on circulating T follicular cells in humans [31]. Regarding Tfr cells, a reduced in vitro suppressive function of cells isolated from autoimmune patients was reported, and both increased or decreased numbers of circulating Tfr cells were observed in different settings [31, 32]. Overall, these studies indicate that autoimmune patients show increased numbers or overactivated Tfh cells and possible quantitative or qualitative alterations in the Tfr compartment, thereby suggesting the implication of T follicular cells in antibody-mediated autoimmunity in humans. However, a better understanding of the biological role of Tfr and Tfh in different disease settings is needed to clarify the apparent contradictions found among different studies. This will be essential for a rational modulation of T follicular cells with therapeutic purposes in autoimmune patients.

## REGULATION OF FOREIGN-REACTIVE ANTIBODY RESPONSES BY Tfr CELLS

The differentiation of Tfr cells is triggered by an antigenic stimulus and thus most of the studies evaluating the biology of Tfr cells, including those evaluating the Tfr role in autoimmunity, involved the experimental infection of mice or their immunization with foreign antigens. In these works, it became clear that the absence of Tfr cells leads, not only to the emergence of self-reactive antibodies, but also to altered responses to the foreign antigen used to trigger the immune response.

As discussed above, although the immune system can be manipulated to elicit foreign-specific Tfr cells (particularly in the absence of competition with thymic Treg cells) [33], under physiological conditions the majority of Tfr clones arise from Foxp3^+^ thymic Tregs and do not recognize the non-self-immunizing antigen [11–13, 23, 25]. However, whether and in which conditions the Tfr regulation of antibodies against foreign antigens occurs via cognate interactions is not yet clear. In fact, this seems to be dispensable as the capacity of Tfr cells to regulate the effector functions of bystander cells with different specificity has already been demonstrated. In an *in vitro* antigen-specific suppression assay, Tfr cells sorted from mice immunized with NP-OVA or NP-hen egg white lysozyme (HEL) and then cultured, in the presence of NP-OVA, with Tfh and B cells from mice immunized with NP-OVA suppressed B cell class switch recombination and Tfh proliferation independently of the immunizing antigen [44].

Regardless of the specific mechanisms involved, several studies show that Tfr cell manipulation impacts features of the antibody response against foreign antigens that are critical for vaccine safety and efficacy. These include the magnitude of the GC response and antibody titres, the affinity of the antibodies and the nature of isotype switch.

### Regulation of the magnitude and specificity of the GC reaction

The initial studies describing Tfr cells reported larger GCs and increased expansion of GC B cells in mice lacking Tfr cells upon immunization, suggesting that this population controls the magnitude of the GC response [11–13]. However, differences were found regarding the impact on the specificity of the response towards the foreign antigen, evaluated either by assessing the levels of GC B cells that recognize the immunizing antigen or the titres of antigen-specific antibodies. While Chung *et al.* and our group observed increased specific responses towards the immunizing antigens in mice devoid of Tfr cells (immunized with NP-KHL in CFA and OVA/alum, respectively) [11, 13], Linterman *et al.* [12] found a decreased specific response against the foreign antigen upon SRBC immunization, despite a larger expansion of Tfh and GC B cells.

More recently, the works using murine models for the conditional depletion of Tfr cells also provided conflicting results on these issues. Most studies using Foxp3^cre^ mice with floxed Bcl6 reported minor changes in the overall GC reaction of Tfr-deficient mice, but all reported qualitative alterations in the specific response towards the foreign antigens. Wu *et al.* [29] observed that the levels of GC B cells evaluated 10 days after a primary immunization with SRBC were identical to control mice and obtained similar results with other types of immunization and at different time-points. However, despite similar Tfh and GC B cell responses, the titres of anti-SRBC IgG were significantly decreased in KO mice, while the levels of specific IgA were increased. The same pattern was observed when mice were immunized with NP-KLH/alum [29]. In a similar mouse model, an OVA prime-boost immunization also resulted in comparable levels of total GC B cells with a reduction in antigen-specific GC B cell from around 20% to 5% when compared with Tfr-sufficient mice [28]. In an influenza-infection model, Botta *et al.* did not observe a similar decrease on antigenic-specific GC B cells or Tfh cells but found a striking impact on the differentiation of CD138^+^ ASCs [23]. The overall ASC levels were increased in the absence of Tfr cells, but the frequency of CD138^+^ ASCs specific for the influenza virus-nucleoprotein was significantly reduced [23]. Another study using influenza infection in Bcl6^fl/fl^Foxp3^cre^ mice also found a negative impact of Tfr deficiency on the specific response towards the foreign antigens concomitant with insignificant alterations on the general levels of GC B cell, Tfh, and Treg compartments [45]. In this study, the Tfr-deficient mice had reduced haemagglutinin (HA)-specific GC B cells, reduced HA-specific ASCs in the bone marrow and reduced IgG titres for HA and neuraminidase at 36 days post-infection [45]. In the same line, the memory response seemed to be decreased in the absence of Tfr cells when evaluated through sequential immunization with antigenically distinct influenza strains (that have different HA head domains but share a conserved stalk domain, thus allowing simultaneous evaluation of primary and recall responses) [45]. Moreover, the authors show that Tfr cells shape the antigen-specific B cell repertoire, as BCR mRNA sequencing revealed that Tfr cell-deficient mice used distinct heavy-chain variable genes upon influenza infection and HA immunization compared with control mice [45]. The notion that Tfr cells may have a ‘helper’ function on the GC reaction, important for optimizing the response towards foreign antigens, was also suggested in a lymphocytic choriomeningitis virus infection model in which the IL-10 produced by Tfr cells was shown to contribute for optimal GC development [46]. Another group, using a murine model of peanut allergy and Bcl6^fl/fl^Foxp3^cre^ mice, also found that Tfr cells may play a supportive role over the GC B cells by repressing the development of Tfh cells with an abnormal cytotoxic phenotype [47].

Contrary to what was in general observed in the Bcl6-floxed systems, the murine models in which Tfr depletion is based on CXCR5 expression revealed either an irrelevant or a positive effect of Tfr-depletion over the magnitude of the foreign-antigen-specific response. In the Cxcr5^fl/fl^Foxp3^cre-ERT2^ model, a reduction in Tfr levels to 50–60% neither impact the size of GC, as evaluated by microscopy, nor the numbers of GC B cells and Tfh, either after NP-KLH/alum immunization or influenza infection [38]. However, in the Tfr-DTR murine model, where CXCR5^+^Foxp3^+^ Tfr cells can be depleted through administration of diphtheria toxin (thus allowing to interfere in the response at different stages of the GC), total and antigen-specific IgG levels were increased compared with control mice when depletion was induced at an early stage of the GC response [30]. Moreover, different from what was observed by Lu *et al*. [45] in the influenza model regarding the memory phase, Tfr-depletion during the primary immunization led to increased antigen-specific antibody responses upon boosting, 30 days later [30]. In this Tfr-DTR murine model, contrasting with the results obtained with early Tfr depletion, no differences were found in GC B cells nor in NP-specific IgG levels when Tfr cells were depleted after the formation of GCs (10–14 days after primary NP-OVA immunization).

Although the results diverge in different models, these studies demonstrate that interfering with Tfr cells may influence both the magnitude and the specificity of the GC-dependent humoral response against foreign antigens. The fact that the impact varies according to the time when Tfr cells are specifically deleted, suggests that different aspects of the response are controlled at specific stages of the GC reaction [21, 30, 48]. It seems that the absence of Tfr cells at an early stage of the GC development has a major impact on the magnitude of the response, as shown in the Tfr-DTR model [30] and corroborated by studies in murine models where total Tregs are briefly depleted at an early phase of the induced response [48]. These observations raised the hypothesis that Tfr cells may play a critical regulatory role in the B cell follicle even before the emergence of the GC [21, 37]. For the purposes of vaccine design, it will be important to elucidate in which circumstances a less stringent early Tfr-suppression can promote the production of antibodies specific to the immunizing antigens without compromising the specificity of the response. Further investigation is also needed to clarify how and at which stages Tfr cells control other features of the GC reaction that impacts the specific response to foreign antigens, such as the differentiation of GC-derived plasma cells or the resolution of the GC reaction. In fact, the divergent results observed in the mentioned studies indicate that Tfr cells may act differently depending on the type of immunization or infection. For instance, the kinetics of Tfr emergence is delayed in the context of influenza infection when compared with vaccination with a protein or allergic sensitization [23, 28, 30]. This diversity on the development kinetics of Tfr cells must be taken in consideration when studying different adjuvants or antigenic formulations in the context of vaccine design.

### Regulation of affinity maturation

A major function of GC reactions is the generation of plasma and memory B cells with the ability to produce high-affinity antibodies. This process relies on the somatic mutation of the genes encoding B cell receptors, followed by the selection of B cell clones that bind the target antigen with higher affinity [49].

In line with the notion that Tfr effector mechanisms have a suppressive nature over the GC reaction, initial studies reported that antibodies produced in the absence of Tfr cells show increased high-affinity antibodies towards an immunizing hapten [13]. Later, Fu *et al.* [27] immunized Bcl6^fl/fl^Foxp3^Cre^ KO mice with NP-KLH and although affinity maturation (evaluated by the ratio of anti-NP4/NP29 antibodies) had no clear alterations in a primary response, it was significantly increased either for IgG or IgG2a when the response was boosted. More recently, these results were corroborated in another study using also the Bcl6^fl/fl^Foxp3^Cre^ system, in which KO mice developed less OVA-specific GC B cells upon OVA prime-boost immunization (although total GC B cells were at comparable levels) but the few OVA-specific cells had a significantly higher affinity, as determined by OVA gMFI by flow cytometry [28].

However, other studies revealed a positive role of Tfr cells on the affinity maturation process [29, 30, 45, 50, 51]. In a model where naive CD4^+^ T cells with Bcl6‐deficient or Bcl6‐sufficient CD25^+^ Treg cells were co‐transferred into T‐cell‐deficient mice, IgA produced in Peyer’s patches from mice lacking Tfr cells had lower affinity and diversity [50]. Sage *et al.* [51] also reported that the immunization of mice transferred with CTLA-4-deficient Tfr cells shows increased NP-specific IgG titres but with a lower NP2/NP16 ratio, suggesting lower affinity. The same profile was observed in the Tfr-DTR mice model when CXCR5^+^ Tfr cells were depleted after immunization with NP-OVA in addavax adjuvant (but before the GC formation) and then boosted with NP-OVA in saline buffer 30 days later. After boosting, the NP-specific antibody titres were higher in mice that had been Tfr-depleted before GC initiation at priming, but showed lower NP2/NP16 ratio [30]. In the same line, using a DNA prime-protein boost vaccine strategy to induce antibody response against the human immunodeficiency virus (HIV)-1 envelope glycoprotein gp120, Wu *et al.* [29] observed a significant decrease in the avidity of anti-gp120 IgG in Bcl6^fl/fl^Foxp3^cre^ mice, as determined by enzyme linked immunosorbent assay (ELISA) with a chaotropic reagent displacement method. Using similar ELISA methods and also in Bcl6^fl/fl^Foxp3^cre^ mice, Lu *et al*. [45] found reduced avidity of anti-HA IgGs in Tfr cell-deficient mice 36 days after influenza virus infection.

These studies indicate that, at least in some conditions, Tfr cells may be important for the emergence of clones with higher affinity for the foreign antigens. A possible explanation is that Tfr cells, by limiting the help provided by Tfh cell to GC B cells, impose a higher level of competition on emergent B cell clones, thus restricting the positive selection to clones with higher affinity, while limiting the general expansion of B cells in the GC.

### Regulation of B cell class-switch

Tfr cells have been shown to impact B cell isotype switch *in vitro*. In co-cultures with Tfh and B cells, Tfr cells suppress class switch recombination to IgG1, and this effect is reduced when Tfr cells are CTLA‐4‐deficient [51]. Also, in an *in vitro* model to evaluate the effect of Tfr cells in the class-switch recombination of autoreactive B cells, Clement *et al.* [30] co-cultured Tfh and Tfr cells sorted from MOG_35–55_-immunized Foxp3^GFP^ mice together with B cells isolated from naive IgH^MOG^ mice (that develop MOG-specific B cell receptors) in the presence of recombinant MOG. In these conditions, IgH^MOG^ B cell-expansion and class-switch recombination induced by Tfh cells was suppressed in the presence of Tfr cells.

In these systems, the suppression of isotype switch from IgD/IgM to IgG may simply reflect a general suppression of the GC response and not a specific reduction of a particular isotype. However, *in vivo* studies reported that Tfr cell deficiency may impact differently over different antibody isotypes. Gonzalez-Figueroa *et al.* [28] analysed baseline titres of different subclasses in Bcl6^fl/fl^Foxp3^Cre^ mice and observed higher IgG1, IgE and IgA in mice lacking Tfr cells, but a reduction in IgG3. However, in a similar murine model, Fu *et al.* [27] found lower levels of NP29-specific IgG1 in KO mice after immunization with NP-KLH in CFA, but significantly higher levels of IgG2a, IgG2c and IgA antibodies and comparable levels of IgG2b, IgG3 and IgM (although Tfh cells numbers were not changed). The production of IgG2c was also enhanced in KO mice upon influenza infection, although total antiviral IgG levels were unchanged [27]. Interestingly, in these settings the IFN-γ expression in CD4^+^Foxp3^−^Bcl6^+^ Tfh cells was significantly increased, providing a possible explanation for the differential isotype switch [27]. Altered cytokine production in Tfh cells in the absence of Tfr cells was also observed by another group upon SRBC immunization and following an HIV-1 gp120 DNA prime-protein boost protocol, with PD1^hi^ Tfh cells from Tfr-deficient mice expressing higher levels of IFN-γ, IL-10 and IL-21 [29]. In a recent study, fibrinogen-like protein 2 (Fgl2) was identified as a soluble effector mechanism secreted by Tfr cells that also regulate Tfh cytokine production (in addition to directly act over GC B cells) and appears to have a specific role in controlling type 2 antibody responses [29,42].

Due to its importance for allergic responses, the regulation of IgE class switching by Tfr cells has been capturing major attention. Allergen-specific high-affinity IgE may arise by sequential switching of affinity-matured IgG1^+^ B cells [52] and underlies many allergic diseases. IL-4 production by Th2 cells has long been linked to the induction of IgE antibodies [52] but Tfh cells, that also produce IL-4, have been found to play a crucial role in IgE responses as well [53–57]. Recently, a novel Tfh subset—Tfh13—has been identified as being crucial for inducing anaphylactic IgE production to allergens [30, 52]. Tfh13 cells produce IL-4 and IL-13, express Bcl6 and Gata3, and are transcriptionally different from Th2 or IL-4-expressing Tfh2 cells. Tfh13 cells are detected in sensitized mice only when high-affinity IgE is present and a cell population equivalent to mouse Tfh13 was identified in human allergic patients [52]. Mouse models lacking Tfr cells (Bcl6- or CXCR5-floxed and Foxp3^Cre^) revealed the major role of Tfr cells in controlling IgE responses in different settings. In an house dust mite (HDM) mouse model, Tfr cells generated *in vivo* were able to suppress Tfh13-B cell cocultures *in vitro*, inhibiting Tfh cell proliferation and B cell switch both to IgG1 and IgE classes [30]. *In vivo*, the deletion of Tfr cells in HDM sensitized mice, although did not impact the overall levels of the lymphocyte populations of the GC reaction, led to a slight increase in IgE plasma cells and significantly higher levels of total and HDM-specific IgE [30]. Moreover, increased inflammation in the lungs with granulocytes and eosinophils infiltration was observed compared with control mice [30]. In another model of Tfr-deficient mice, immunization protocols with intra-peritoneal OVA/alum or intra-gastrical peanut sensitization resulted in increased levels of total and antigen-specific IgE, but not IgG [28]. When re-challenged intravenously, Tfr-deficient mice showed anaphylactic reactions, succumbing shortly after challenge, and presenting elevated levels of histamine in circulation [28]. Importantly, the authors of this study identified a novel Tfr cell effector mechanism mediated by neuritin. Neutitin is a protein produced by Tfr cells that directly targets B cells, dampening IgE class switching, suppressing the accumulation of early plasma cells in GCs and hindering the development of autoantibodies [28]. In humans, B cell IgE class switching has also been shown to be inhibited by IL-10-producing T follicular cells, another recently described T follicular cell subset with similarities to mouse Tfr cells that lack Foxp3 expression [58].

Overall, the different reports show that the Tfr cells can inhibit the production of antibodies with different isotypes. It is not yet possible to establish whether there is a preferential isotype switching influenced by Tfr cells, or simply a preferential reduction of the isotypes that are being predominantly produced at the time of Tfr suppression.

## CONSIDERING Tfr CELLS IN VACCINE DEVELOPMENT

**Figure 2:**
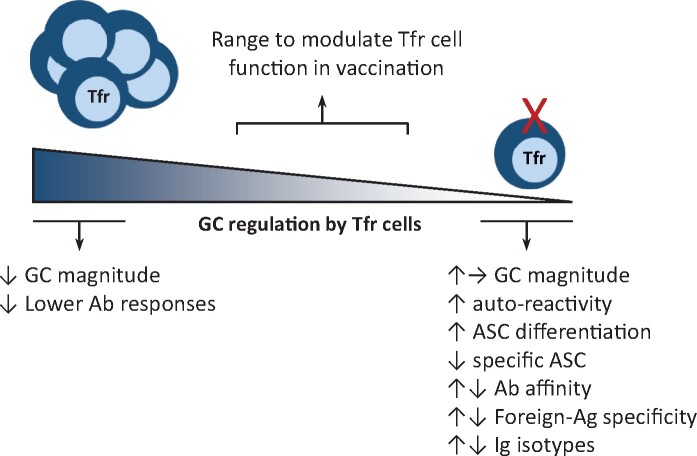
modulation of Tfr cells for improved vaccine efficacy. Reducing the Tfr cell suppressive function over the GC reaction while promoting the differentiation of Tfh cells is a rational strategy to enhance GC-dependent antibody responses in vaccination. However, the induction of dysfunctional Tfr cells or highly reduced Tfr cell levels may lead to increased autoreactivity, altered immunoglobulin isotypes and possible lower antibody affinity, thereby resulting in detrimental outcomes. The challenge for modulating Tfr cells in the context of vaccine design will be to find how and to what extent their suppressive functions can be restrained while preserving a stringent selection of foreign-reactive high-affinity B cell clones.

Neutralizing antibodies are a major hallmark of the most effective vaccines and thus the T follicular cell subsets specialized in controlling GC reactions readily became obvious targets for improving vaccine responses. Restraining Tfr cells while promoting Tfh cell differentiation seemed to be a logical strategy to enhance long-lived high-affinity antibody responses, which depend on GC reactions. In this sense, different adjuvants have been evaluated in their capacity to alter the Tfh:Tfr ratio [59]. However, it is now clear that the participation of Tfr cells in GC reactions is critical to focus the antibody specificity towards the immunizing antigen while avoiding the emergence of self-reactive antibodies. The need to preserve this control must be taken into account particularly for vaccination of target populations with a tendency to develop self-reactive antibodies, such as autoimmune patients or the elderly. In addition, at least in some circumstances, Tfr cells seem to play a positive role in the development of high-affinity antibodies, by guaranteeing a high stringency in the Tfh-dependent selection of B cell clones. Therefore, in the context of vaccine development, the challenge for modulating the GC reaction will be to achieve the right balance between the factors that enhance and control the GC reaction upon immunization to assure the induction of high-quality immune responses while avoiding autoreactivity (Fig. 2).

Rational modulation of Tfr cells in vaccine design will require a better knowledge about the developmental cues driving Tfr differentiation and their effector mechanisms (Fig. 1). It is known that Tfr cell differentiation requires the priming by DCs [44], which involves TCR triggering and costimulatory signals through CD28 and ICOS [12, 60], and that B-cell interactions are needed for completing their terminal differentiation into mature GC Tfr cells [12, 22, 44]. However, the soluble and contact-dependent factors that drive Tfr cell differentiation are still poorly characterized. The cytokines IL-2 [23, 61] and IL-21 [41, 62, 63], as well as PD-1 [60] and CTLA-4 [48, 51] signalling, have been shown to dampen Tfr cell differentiation and are potential targets for controlling Tfr maturation. Regarding Tfr cell effector mechanisms, CTLA-4 [48, 51] and, more recently, neuritin [28] and Fgl2 [42] are the molecules that have been better characterized regarding Tfr function. Therefore, CTLA-4, neuritin and Fgl2 are potential targets for the modulation of Tfr function and, consequently, for GC regulation. Additional regulatory mechanisms established for other Treg subsets—such as IL-10, TGF-β and granzyme B—were also proposed [46, 64]. Molecular targets are thus starting to emerge and their modulation in the vaccine context, particularly in individuals identified as low-responders, may be envisaged in the future. For instance, the inclusion of Tfr-targeting molecules in vaccine formulations or the administration of Tfr modulators prior or concomitantly to vaccine inoculation, either locally or systemically, could be considered. However, the practicality of these strategies is yet to be tested and several steps must be taken in pre-clinical studies to evaluate their effectiveness and potential side effects. Given reports showing that some Treg and Tfr cells can lose Foxp3 expression, acquiring characteristics closer to Tfh cells [14, 34–36], it remains to be shown whether such Tfr instability may need to be considered in rational vaccine design.

At present, the development of adjuvants or immunizing strategies promoting the induction of Tfh cells and stronger GC reactions is already an integral part of the rational process for vaccine development. Disclosing the Tfr developmental cues and effector mechanisms, together with a greater understanding of the Tfr cell role in controlling the diverse features of the GC reaction, will provide the necessary rationale to integrate the modulation of Tfr cells in vaccine design, thus contributing for safer and more effective vaccines.

## FUNDING

This work was funded by UTA-EXPL/NPN/0082/2019, EJPRD/0003/2019 and LISBOA-01-0145-FEDER-007391, projeto cofinanciado pelo FEDER através POR Lisboa 2020—Programa Operacional Regional de Lisboa, do PORTUGAL 2020, e pela Fundação para a Ciência e a Tecnologia to L.G.; and by Fundação para a Ciência e a Tecnologia—PTDC/CVT-CVT/31840/2017 to A.P.B.

## AUTHORS’ CONTRIBUTIONS

A.P.B. and L.G. jointly contributed to writing, reviewing and editing the manuscript.

## CONFLICT OF INTEREST STATEMENT

The authors have no conflicts of interest to declare.
